# Attachment style and mental health during the later stages of COVID‐19 pandemic: the mediation role of loneliness and COVID-19 anxiety

**DOI:** 10.1186/s40359-022-00767-y

**Published:** 2022-03-14

**Authors:** Laura Vismara, Loredana Lucarelli, Cristina Sechi

**Affiliations:** Department of Pedagogy, Psychology and Philosophy, University of Cagliari, Cagliari, Italy

**Keywords:** COVID-19 pandemic, Attachment styles, Loneliness, COVID-19 related anxiety, Mental health problems

## Abstract

**Background:**

An insecure attachment style is an important risk factor for psychological problems. The aim of this study was to use Bartholomew and Horowitz’s model (Bartholomew and Horowitz in J Pers Soc Psychol 61(2): 226, 2019) to test a sample of Italian individuals to determine the mediation role of loneliness and COVID-19-related anxiety symptoms in the relationship between attachment styles and mental health issues in the context of the pandemic.

**Method:**

A cross-sectional research study was conducted using a sample of 330 Italian participants (82.1% women; mean age = 34.3 years; SD = 13.16) who completed online self-reported measures of attachment styles (RQ), loneliness (RULS), COVID-19-related anxiety symptoms (C-19ASS) and mental health problems (GHQ-12). Serial mediation analyses were performed, and bootstrap tests were included.

**Results:**

Our results supported the hypothesized model with respect to each attachment style (*p* < 0.001). In particular, insecure attachment styles predict mental health problems both directly and indirectly, through loneliness and COVID-19-related anxiety symptoms. In addition, loneliness directly influences mental health problems and also mediates the relationship between insecure attachment styles and COVID-19-related anxiety symptoms which, in turn, positively predict mental health problems.

**Conclusions:**

Our findings reinforce the importance of attachment in people’s processes of adapting to experiences during the coronavirus pandemic. The study makes an important contribution to developing effective prevention and intervention strategies to support people’s wellbeing in the context of the pandemic.

## Background

Experiencing the COVID-19 pandemic has generated a sense of uncertainty and distress that has understandably activated the attachment system, which is aimed at seeking proximity to others of significance in order to gain their support under stress conditions. Indeed, one of the core concepts of Bowlby’s [10–12] attachment theory is that a threating condition, such as the COVID-19 pandemic, automatically triggers the attachment system.

According to the quality of their attachment style, people differ in terms of their internal representation of the self and others: *securely attached individuals* have a positive model of the self and others, and they value relationships; *fearfully attached individuals* are characterized by negative representations of both the self and others, and, although dependent on others, they are worried about intimacy; *preoccupied attachment* is characterized by a negative model of the self and a positive model of others, with individuals consequently clinging to others due to being anxious about abandonment; finally, *dismissive attachment* presents a positive representation of the self and a negative representation of others, meaning that dismissive persons appear self-assured and discredit relationships [5, 14, 23]. Insecure attachment has been found to predispose people to higher vulnerability to stress, which can increase the risk of developing poor mental health symptoms, in addition, insecure attachment impairs the capacity to seek beneficial support, influencing responses to life events [2, 33, 43]. Several scholars have also investigated the variables that may elucidate the association between attachment and mental health problems [32, 38, 45, 48, 56]. We believe that loneliness and COVID-19-related anxiety symptoms may be among the mechanisms that can explicate the relationships between attachment and health in the context of the COVID-19 pandemic.

### Attachment and loneliness

As noted by Mikulincer and Shaver [53] the scarceness quality of the relationships of insecurely attached individuals may give rise to feelings of loneliness. Loneliness may be defined as the subjective experience of poor social interactions [3] and appears to be a consequence of the painful feeling of an incongruity between the individual’s desired and perceived relationships [17]. Most of the attachment scholars have examined the link between insecure attachment and loneliness through the study of social skills [35, 40, 44]. People who refer to feeling lonely show limited interpersonal capacities that are essential for initiating and nourishing intimate relationships [6], or they assume themselves to be lacking in those capacities [40]. Securely attached individuals consistently display higher social skill levels [16]. Hence, insecure attachment may be considered a predictor of loneliness, whereas secure attachment promotes good relationships with others [46]. Attachment styles affect how individuals represent themselves and others; these representations influence how lonely they feel. Correspondingly, loneliness may increase vulnerability to mental health problems [15, 54, 56].

### Loneliness and the COVID-19 pandemic

During the COVID-19 pandemic, people with significant feelings of loneliness refer to more negative experiences connected with the situation [36, 42, 49]. In this specific context, loneliness may be exacerbated by the anxiety of losing interpersonal supports or being alone during a time of uncertainty regarding health and safety [27]. Quite a few studies have demonstrated that loneliness has a negative effect on psychological health [50, 62]. As a consequence of the pandemic’s forced isolation, the emotioanl burden of loneliness has spread out in community samples [4, 27, 31], this condition increases the likelihood of developing psychopathological problems that need more in-depth analyses [41]. Therefore, the present study, within an attachment theory perspective, posits loneliness as a possible affective and cognitive mediator that may increase the risk of developing COVID-19-related anxiety and mental health problems in the context of the pandemic.

### The COVID-19 pandemic and mental health

The COVID-19 pandemic has undeniably led to increases in psychological problems of different types and severities [18, 26, 39, 59]. However, few investigations have addressed mental health issues and feelings of loneliness in the general population due to both the pandemic and the adopted control measures, such as social distancing [4, 21], lockdown [34, 70] and quarantine [60]. Even fewer studies have addressed the relationship between attachment style and mental health difficulties during the COVID*-*19 pandemic despite the well-known association between attachment insecurity and the onset of psychological problems [52].

### Aim and hypotheses

This study is aimed at understanding how attachment styles operate through loneliness to influence mental health. The recognition of this mechanism may improve the efficacy of preventing psychological distress in the specific context of the COVID-19 pandemic. Thus, we propose a dual mediation model of the relationship between attachment style and mental health problems. Based on the research reviewed, we assumed that insecure attachment styles are associated with an increase in mental health issues and that loneliness and COVID-19 anxiety symptoms are possible mediators of this relationship. We assumed that insecure attachment styles can lead to difficulties in relating to others and regulating feelings, provoking loneliness. We also assumed that experiencing loneliness during the COVID-19 pandemic (which itself is predicted by attachment styles) increases the probability of suffering from COVID-19-linked anxiety symptoms when it is not possible to rely on perceived social and psychological support for an effective response to the pandemic. Lastly, we assumed that COVID-19-related anxiety symptoms would predict amplified mental health problems because individuals who experience several adverse outcomes during the COVID-19 pandemic are likely to feel they cannot cope with the encompassed stressful events, therefore experiencing elevated mental health problems.

The specific research hypotheses are as follows.

#### **Hypothesis (1)**

Insecure attachment styles are positively associated with the individual’s mental health problems;

#### **Hypothesis (2)**

Loneliness plays a mediating role between attachment style and mental health problems;

#### **Hypothesis (3)**

COVID-19 anxiety syndrome plays a mediating role between attachment style and mental health problems;

#### **Hypothesis (4)**

Loneliness is positively associated with COVID-19 anxiety syndrome and plays a chain mediating role between attachment style and mental health problems.

## Method

### Participants

This cross-sectional study was performed in Italy during the COVID‐19 Pandemic via an internet survey from the 1st of November 2020 to the 28^th^ of February 2021. A convenience sampling method was used to recruit participants. The inclusion criteria were: (a) 18 years old or above and (b) living in Italy. The participants were recruited online; participants were invited to take part in the research through a brief advertisement posted on Italian platforms, including social media and social groups inviting them to share the link amongst their friends.

The participants answered anonymously by filling up an informed consent letter in the first section of the e-survey. A total of 330 participants (82.1% women; mean age = 34.33 years [SD = 13.17; range 18–63 years]) was enrolled for this investigation. All participants were Caucasian. Most of the sample was educated at university level (53.3%), employed (61.8%), married or co-habiting (74.2%). Sociodemographic characteristics are summarized in Table 1.

**Table 1:** Demographic and socioeconomic sample’s characteristics

Variable	(N = 330) N (%)
*Age* (years)	
18–25	124 (37.6%)
26–35	86 (26.1%)
36–50	61 (18.5%)
≥ 51	59 (17.9%)
*Gender*	
Men	59 (17.9%)
Women	271 (82.1%)
*Educational level*	
Middle school	37 (11.2%)
High school	117 (35.5%)
University degree	176 (53.3%)
*Marital status*	
Unmarried	85 (25.8%)
Married	245 (74.2%)
*Occupation*	
Student	95 (28.8%)
Employed	204 (61.8%)
Unemployed	31 (9.4%)
*Geographical area*	
Northern Italy	596 (78.3%)
Central Italy	49 (6.4%)
Southern Italy and Islands	116 (15.2%)
Northern Italy	34 (10%)
Central Italy	86 (26%)
Southern Italy and Islands	210 (64%)

### Measures

#### Adult attachment styles

To evaluate attachment styles, we utilized the Relationship Questionnaire (RQ; [5]). The RQ is a single-item measure, consisting of four short distinct sections illustrating the secure, dismissing, preoccupied and fearful attachment styles.

Specifically, there are two parts, RQ 1 and 2. In the first part, participants were asked to select a paragraph-long description that best described them without providing a numerical rating. An example statement for RQ1 is as follows: Fearful attachment: “I am uncomfortable getting close to others, I want emotionally close relationships, but I find it difficult to trust others completely, or to depend on them. I worry that I will be hurt if I allow myself to become too close to others”. In the second part, RQ2, participants are invited to rate their agreement with each prototype on a 7-point Likert scale (from “1 = disagree strongly” to “7 = agree strongly”). Regarding psychometric properties, internal consistency cannot be calculated. The retest reliability for RQ was previously evaluated as being in the range of 0.74–0.88 [37]. The RQ scores have shown good agreement with observer-based ratings of self-reported ratings for interpersonal problems, and dimensional measures of attachment [25]. The RQ has also demonstrated good convergent and discriminant validity across cultures [64].

### Loneliness

To evaluate feelings of loneliness, we utilized the Revised UCLA Loneliness Scale (RULS; [61]. It is a 20-item Likert scale and assesses individuals’ level of loneliness as characterized by a difference between real and desired social contact (i.e., “How often do you feel alone?”, “How often do you feel isolated from others?”). Participants are invited to assess how often they feel the way illustrated in each item. The questionnaire is scored using a 4-point Likert scale (from “1 = never” to “4 = often”). Higher scores suggest greater feeling of loneliness. Scores ranged from 20 to 80. Higher scores showed an increased severity of loneliness. It has been widely used in previous research and has shown consistently high internal consistency, with a coefficient α ranging from 0.89 to 0 0.94 and test–retest reliability over a 1-year period (r = 0.73) [61]. In the present study, Cronbach for the whole scale was *α* = 0.81.

### COVID-19 anxiety

To assess the anxiety symptoms related to COVID-19 we utilized the COVID-19 Anxiety Syndrome Scale (C-19ASS; [55]. It is a new 9 item Likert scale and evaluates aspects of the anxiety syndrome related to COVID-19. Two factors of the scale include (1) perseveration (C-19ASS-P), with items concerning checking (e.g., symptoms of COVID-19), worrying (e.g., investigating symptoms of COVID-19) and threat monitoring (e.g., paying close attention to others showing potential symptoms of COVID-19) and (2) avoidance (C-19ASS-A) (e.g., of public transport because of the fear of contracting COVID-19). Participants are invited to assess how often they experience each characteristic of the anxiety syndrome. The questionnaire is scored using a 5-point scale (from “0 = not at all” to 4 = nearly every day over the last 2 weeks”). Scores range between 0 and 36, with higher scores indicative of increased levels of the anxiety syndrome. Both the C-19ASS-P (α = 0.86) and the C-19ASS-A (α = 0.77) demonstrated acceptable levels of reliability [55]. In the present study, the overall C-19ASS had a reliability of α = 0.83 while the C-19ASS-P had α = 0.81 and the C-19ASS-A had α = 0.74.

### Mental health problems

To assess mental health problems, we applied the General Health Questionnaire (GHQ-12; [22], Italian version by Politi et al. [57]). It consists of 6 positively worded items (e.g. “Felt capable of making decisions about things”), and six negatively-worded items (e.g. “Lost much sleep over worry”) and evaluates mental distress in the common people. Participants are invited to rate how often they experience each behavior or symptom in the past 2 weeks. The questionnaire is scored using a 4-point scale (from “0 = not at all” to “3 = much more than usual”). Higher scores suggest higher levels of mental distress. Internal consistency reliabilities of the global score ranged from 0.79 to 0.91 [66, 71]. In the present study, Cronbach for the whole scale was *α* = 0.81.

### Data analyses

The statistical analyses were conducted with IBM SPSS Statistics program, 24 version. We primarily investigated the descriptive statistics of the study variables and relationships between the variables using Pearson correlation analysis. Then, we created four separate models for each of attachment styles; Model A: Secure; Model B: fearful; Model C: Preoccupied; and Model D: Dismissing.

**Fig. 1:**
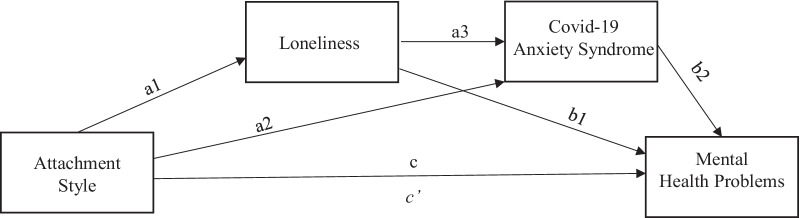
Illustration of a serial multiple-step indirect effect, attachment style (predictor variable) is hypothesized to effect directly and indirectly on Mental health problems (outcome variable) through Loneliness (Mediator 1) and Covid-19 Anxiety Syndrome (Mediator 2) (PROCESS Multiple Mediation Model 6; Hayes, 2018) [28]. (c) A direct effect of the impact of Attachment Style on the Mental Health Problems. (a1, b1) An indirect effect of the Attachment Style on Mental Health Problems, including Loneliness. (a2, b2) An indirect effect of the Attachment Style on the Mental Health Problems, including Covid-19 Anxiety Syndrome. (a1, a3, b2) An indirect effect of the Attachment Style on the Mental Health Problems, including Loneliness and Covid-19 Anxiety Syndrome. (*c′*) A direct effect of the Attachment Style on the Mental Health Problems, taking account of the impact of both mediators

Moving on to the verification of the four models, it was important to check the mediation role of feelings of loneliness (mediator 1) and COVID-19 Anxiety (mediator 2), as well as the joint influence of both mediators on the relationship between attachment style and mental health problems. Serial mediation analyses were performed using model six of Hayes’ PROCESS macro [28] in order to run the planned analysis of mediation models A, B, C and D, outlined in Fig. 1. Furthermore, bootstrap analyses with bias-corrected confidence estimates on a 95% confidence level [58] were used in order to test significance of total, direct, and indirect effects. Bootstrap estimates were based on 10,000 bootstrap samples.

## Results

### Descriptive analysis and correlations

The descriptive and correlation analyses can be seen in Table 2. Correlations revealed that secure attachment style was negatively linked with feelings of loneliness, COVID-19 Anxiety Syndrome, and mental health problems. Contrariwise, fearful and preoccupied attachment styles were positively linked with feelings of loneliness, COVID-19 Anxiety Syndrome and mental health problems. Lastly, dismissing attachment style was positively correlated with feelings of loneliness and mental health problems. No significant relationships were found between dismissing attachment style and COVID-19 Anxiety Syndrome.

**Table 2:** Means, standard deviations and correlations for the study variables

	Mean	SD	1	2	3	4	5	6	7	8	9	10
1.Gender (0 = man; 1 = woman)	_	_	_									
2.Age	34.38	13.16	.036	_								
3.Educational level	_	_	− .079	.144**	_							
4.RQ-secure attachment	3.32	1.72	.045	.028	− .085	_						
5.RQ-RQ-fearful attachment	2.78	1.54	− .013	− .021	− .036	− .083	_					
6. RQ-preoccupied attachment	3.13	1.76	.017	− .067	− .025	− .021	.269***	_				
7. RQ-dismissing attachment	3.90	1.75	− .084	.008	.017	− .206***	− .075	.141*	_			
8.RULS	2.09	0.36	− .062	− .059	.036	− .457***	.180**	.312***	.168**	_		
9. C-19ASS	2.75	0.72	− .098	− .104	− .056	− .215***	.201***	.146**	.022	.149**	_	
10.GHQ-12	1.65	0.49	− .100	− .112	− .013	− .155**	.192**	.167**	.139*	.391***	.240***	_

*RQ* Relationship Questionnaire, *RULS* Revised UCLA Loneliness Scale, *C-19ASS* COVID-19 Anxiety Syndrome Scale, *GHQ-12* General Health Questionnaire

*p <.05; **p <.01; ***p <.001

### Mediation analyses

#### Model A (secure attachment)

As shown in Table 3, results showed that secure attachment negatively predicted loneliness (*B* = − 0.45, *t* = − 9.04, *p* < 0.001) and COVID-19 Anxiety Syndrome (*B* = − 0.18, *t* = − 3.00, *p* < 0.05). Loneliness positively predicted COVID-19 Anxiety Syndrome (*B* = 0.13, *t* = 2.26, *p* < 0.05). Both loneliness and COVID-19 Anxiety Syndrome positively predicted mental health problems (*B* = 0.37, *t* = 6.61, *p* < 0.001 and *B* = 0.19, *t* = 3.58, *p* < 0.01, respectively). Finally, the negative and significant direct effect of secure attachment on mental health problems (*B* = − 0.16, *t* = − 2.90, *p* < 0.01) became non-significant (*B* = 0.05, *t* = 0.93, *p* = 0.36) when loneliness and COVID-19 Anxiety Syndrome were included in the model, indicating full mediation.

**Table 3:** Multiple-step mediation analysis on attachment styles (secure; fearful; preoccupied; and dismissing), loneliness, COVID-19 anxiety syndrome and mental health problems

Model A					Model B				
Path estimates	Coeff	SE	BCa 95% CI		Path estimates	Coeff	SE	BCa 95% CI	
			Lower	Upper				Lower	Upper
*a1*	− .45***	.05	− .11	− .07	*a1*	.20**	.01	.02	.06
*a2*	− .18*	.02−	− .12	− .03	*a2*	.15*	− 02	.02	.11
*a3*	.14*	.12	.03	.50	*a3*	.18**	.11	.15	.58
*b1*	.37***	.07	.35	.66	*b1*	.34***	.07	.32	.59
*b2*	.19**	.04	.06	.20	*b2*	.17*	.04	.04	.18
c	− .16*	.02	− .08	− .02	c	.18**	.02	.02	.08
*c′*	.05	.05	− .02	.06	*c′*	.08	.04	− .00	.05

Indirect Effects	Effect	SE	BCa 95% CI		Indirect Effects	Effect	SE	BCa 95% CI	
			Lower	Upper				Lower	Upper
Total	− .21	.03	− .28	− .16	Total	.10	.02	.06	.14
M1	− .18	.03	− .24	− .12	M1	.07	.02	.04	.11
M2	− .06	.02	− .010	− .02	M2	.04	.02	.02	.08
M1&M2	− .02	.02	− .02	− .00	M1&M2	.01	.00	.00	.01

Model C					Model D				
Path estimates	Coeff	SE	BCa 95% CI		Path estimates	Coeff	SE	BCa 95% CI	
			Lower	Upper				Lower	Upper
*a1*	.32***	.01	.05	.10	*a1*	.14*	.01	.01	.05
*a2*	− 15*	.03	.02	.12	*a2*	.07	.02	− .02	.07
*a3*	.17**	.11	.11	.55	*a3*	.21**	.11	.20	.62
*b1*	.32***	.07	.30	.58	*b1*	.34***	.07	.32	.60
*b2*	.17*	.04	.04	.18	*b2*	.17**	.03	.05	.18
c	.23***	.02	.04	.11	c	.17*	.02	.02	.08
*c′*	.10	.02	.− .00	.06	*c′*	.10*	.01	.00	.06

Indirect Effects	Effect	SE	BCa 95% CI		Indirect Effects	Effect	SE	BCa 95% CI	
			Lower	Upper				Lower	Upper
Total	.14	.02	.09	.19	Total	.06	.02	.02	.11
M1	.11	.02	.07	.16	M1	.05	.02	.01	.09
M2	.04	.02	.02	.08	M2	.02	.02	− .01	.06
M1&M2	.01	.00	.00	.05	M1&M2	.01	.00	− .01	.0

M1 = Loneliness; M2 = Covid-19 anxiety syndrome

* *p *<.05; **p <.01; ***p <.001

The analysis of indirect effects (Table 3) showed that the first indirect effect of the impact of secure attachment on the mental health problems with the mediatory role of Loneliness (a1, b1) was statistically significant. As for the second indirect effect, where COVID-19 Anxiety Syndrome constituted a mediator (a2, b2), the result was also statistically significant. Turning to the last indirect effect of the impact of secure attachment on the mental health problems with Loneliness and COVID-19 Anxiety Syndrome as mediators (a1, a3, b2), it was proven that this effect was statistically significant. The total model accounted for a significant amount of variance’ (R^2^ = 0.18) mental health problems and our findings supported the hypothesized model (F (3.326) = 24.47; *p* < 0.001).

#### Model B (fearful attachment)

As shown in Table 3, results showed that fearful attachment positively predicted loneliness (*B* = 0.20, *t* = 3.64, *p* < 0.001) and COVID-19 Anxiety Syndrome (*B* = 0.15, *t* = 2.77, *p* < 0.05). Loneliness positively predicted COVID-19 Anxiety Syndrome (*B* = 0.13, *t* = 2.26, *p* < 0.01). Both loneliness and COVID-19 Anxiety Syndrome positively predicted mental health problems (*B* = 0.34, *t* = 6.50, *p* < 0.001 and *B* = 0.17, *t* = 3.19, *p* < 0.01, respectively). Finally, the positive and significant effect of fearful attachment on mental health problems (*B* = 0.18, *t* = 3.60, *p* < 0.01) became non-significant (*B* = 0.09, *t* = 1.65, *p* = 0.11) when loneliness and COVID-19 Anxiety Syndrome were included in the model, indicating full mediation.

Analysis of the indirect effects in the bootstrapped samples (Table 3) showed that the first indirect effect of the impact of fearful attachment on the mental health problems with the mediatory role of Loneliness (a1, b1) was statistically significant. As for the second indirect effect, where COVID-19 Anxiety Syndrome constituted a mediator (a2, b2), the result was also statistically significant. Turning to the last indirect effect of the impact of fearful attachment on the mental health problems with Loneliness and COVID-19 Anxiety Syndrome as mediators (a1, a3, b2), it was proven that this effect was statistically significant. The total model accounted for a significant amount of variance’ (R^2^ = 0.19) mental health problems and our findings supported the hypothesized model F (3.326) = 25.23; *p* < 0.001).

#### Model C (preoccupied attachment)

As shown in Table 3, results showed that preoccupied attachment positively predicted loneliness (*B* = 0.32, *t* = 6.08, *p* < 0.001) and COVID-19 Anxiety Syndrome (*B* = 0.15, *t* = 2.63, *p* < 0.01). Loneliness positively predicted COVID-19 Anxiety Syndrome (*B* = 0.17, *t* = 2.97, *p* < 0.01). Both loneliness and COVID-19 Anxiety Syndrome positively predicted mental health problems (*B* = 0.32, *t* = 6.07, *p* < 0.001 and *B* = 0.16, *t* = 3.19, *p* < 0.01, respectively). Finally, the positive and significant effect of preoccupied attachment on mental health problems (*B* = 0.23, *t* = 4.31, *p* < 0.001) became non-significant (*B* = 0.10, *t* = 1.79, *p* = 0.07) when loneliness and COVID-19 Anxiety Syndrome were included in the model, indicating full mediation.

Analysis of the indirect effects in the bootstrapped samples (Table 3) further revealed that the first indirect effect of the impact of preoccupied attachment on the mental health problems with the mediatory role of Loneliness (a1, b1) was statistically significant. As for the second indirect effect, where COVID-19 Anxiety Syndrome constituted a mediator (a2, b2), the result was also statistically significant. Turning to the last indirect effect of the impact of preoccupied attachment on the mental health problems with Loneliness and COVID-19 Anxiety Syndrome as mediators (a1, a3, b2), it was proven that this effect was statistically significant. The total model accounted for a significant amount of variance’ (R^2^ = 0.19) mental health problems and our findings supported the hypothesized model (F (3.326) = 25.43 *p* < 0.001).

#### Model C (dismissing attachment)

Finally, as shown in Table 3, results showed that dismissing attachment positively predicted loneliness (*B* = 0.14, *t* = 2.50, *p* < 0.05). Dismissing attachment did not predict directly COVID-19 Anxiety Syndrome (*B* = 0.07, *t* = 1.23, *p* = 0.21). Loneliness positively predicted COVID-19 Anxiety Syndrome (*B* = 0.21, *t* = 3.77, *p* < 0.01). Both loneliness and COVID-19 Anxiety Syndrome positively predicted mental health problems (*B* = 0.34, *t* = 6.59, *p* < 0.001 and *B* = 0.17, *t* = 3.35, *p* < 0.01, respectively). Finally, the positive and significant effect of dismissing attachment on mental health problems (*B* = 0.17, *t* = 3.03, *p* < 0.01) became weaker (*B* = 0.10, *t* = 2.04 *p* < 0.05) when loneliness and COVID-19 Anxiety Syndrome were included in the model. That is, the direct effect remained significant indicating partial rather than full mediation.

Analysis of the indirect effects in the bootstrapped samples (Table 3) further revealed that the first indirect effect of the impact of dismissing attachment on the mental health problems with the mediatory role of Loneliness (a1, b1) was statistically significant. As for the second indirect effect, where COVID-19 Anxiety Syndrome constituted a mediator (a2, b2), the result was statistically non-significant. Turning to the last indirect effect of the impact of dismissing attachment on the mental health problems with Loneliness and COVID-19 Anxiety Syndrome as mediators (a1, a3, b2), it was proven that this effect was statistically significant. The total model accounted for a significant amount of variance’ (R^2^ = 0.19) mental health problems and our findings supported the hypothesized model (F (3.326) = 25.43 *p* < 0.001).

## Discussion

The coronavirus disease is a pandemic event that, beyond physical health, impacts important psychological, social and behavioural outcomes [31, 36]. Hence, it is crucial to determine which factors may offer protections from its potential negative effects. Several studies have demonstrated that attachment insecurity is positively correlated with increased symptoms of poor mental health [20, 51, 52].

The current study is particularly intended to verify the direct association between attachment styles and mental health problems in the context of the COVID-19 pandemic. It was hypothesized that loneliness and anxiety symptoms associated with the pandemic would be important mediators in this association. There is widespread agreement on people’s innate need to gather with others, especially under distressing conditions. Attachment theory provides the theoretical and methodological tools to understand this vital issue. Our results contribute to a fuller appreciation of how and for whom attachment insecurity is associated with mental health symptoms in the context of the current pandemic.

The mediation analyses concerning each attachment style demonstrated that the relationship between attachment styles and mental health problems is partially or even totally explained by perceived loneliness and anxiety symptoms associated with the COVID-19 pandemic. Secure attachment may play a key protective role in reducing mental health problems during the COVID-19 pandemic. Indeed, individuals who are securely attached develop efficacious emotion-regulation abilities, form positive representations of the self and others, and have better perceptions of their psychological wellbeing [51, 52]. As a consequence, they have the capacity to mitigate loneliness, which in turn reduces anxiety symptoms associated with the COVID-19 pandemic, which in turn reduces mental health problems. Our results are consistent with previous research: secure people, within a sensitive and reliable caregiving bond, have developed the ability to regulate emotions and related behaviours, therefore, they face stressful events relying on both others’ support and their own self-confidence [16]. Consequently, securely attached individuals are found to experience higher psychological wellbeing [30, 52, 65], lower perceptions of loneliness [1, 24] and lower levels of anxiety [19, 47, 68].

Fearful and preoccupied attachment styles, in contrast, were confirmed as risk factors for mental health problems in the context of the COVID-19 pandemic. Higher levels of fearful and preoccupied attachment styles are definitely linked to more feelings of loneliness, which in turn increase anxiety symptoms associated with the pandemic, which in turn raise the risk of developing mental health problems. People with preoccupied and fearful attachment styles, who grew up within a compromised parenting environment, actually develop a sense of the self as being worthless and others as being rejecting or malevolent [7], perceive relational distress, and excessively worry about the availability and responsiveness of others, consequently, they may experience heightened feelings of loneliness. Moreover, the lack of a sensitive and responsive caregiver undermines the individual’s regulatory capacity, therefore increasing their risk of developing psychological problems [67], especially in terms of reported worries and anxiety [9], Simonelli et al. 2004]. Being deficient in both personal and interpersonal resources to overcome difficulties, these persons are likely to find the COVID-19 pandemic experience extremely distressing.

Finally, according to the results of the mediation model in the present study, loneliness and anxiety symptoms associated with the COVID-19 pandemic had partially mediated the relationship between dismissing attachment and mental health problems. Indeed, dismissing attachment, despite being significantly linked to mental health problems, showed a weaker direct effect. This weaker relationship is in line with earlier research data that found incongruent results with respect to dismissing attachment [29, 67]. Deeming others as inaccessible and unreliable may be dysfunctional. However, dismissing attachment is associated with a positive representation of the self that may encourage self-isolation, lowering the feeling of loneliness, and may also endorse a sense of self-efficacy that facilitates adjusting to distressing experiences and events, thus decreasing the risk of psychopathology [13], Simonelli et al. 2004].

### Limitations

Results derived from the present study should be interpreted with consideration for several limitations. First, our study was cross-sectional and included only self-report measures. It is not possible to determine the degree to which the findings may be generalized to the wider population and doing so may generate biased estimates of the longitudinal parameters.

Linked to this, the sample had an uneven gender distribution, with more women (82.1%) than men taking part in the study. This distribution is similar to several other online studies addressing a variety of considered constructs [8]. However, it has been demonstrated that gender differences can be relevant. In particular, men seem to be significantly more dismissing than women [63]. Therefore, although the disproportion may be relevant to the specificity of our investigation, caution must be used when generalizing these results to other populations. Future studies with more evenly distributed gender samples could be undertaken to determine whether results generalize across both genders and may consider using longitudinal or experimental research to determine the causal relationships.

To conclude, although our findings were significant, the explained variance was modest. Thus, future studies should include other variables that may provide additional elucidation of the relationship between attachment styles and mental health problems during the COVID-19 pandemic.

## Conclusions

Despite the described limitations, the study provides valuable clinical suggestions that should be carefully considered in the context of the COVID-19 pandemic. The evidence that the attachment style orients the individual’s modality to respond to experiences during the coronavirus pandemic may specifically guide the designs of prevention and intervention programmes aimed at improving mental wellbeing in this specific situation. In particular, because attachment insecurity is closely linked with reported mental health problems through loneliness, diminishing feelings of loneliness may be helpful in reducing psychological malaise in the context of the pandemic. Enhancing the sense of togetherness, endorsing shared values and offering social support during the pandemic may constitute an efficacious way to ameliorate the individual’s response to the pandemic itself.

Our findings emphasize the need for primary and secondary care services to routinely include psychological support to prevent the worsening of people’s health conditions. Special attention should be paid to increasing insecurely attached people’s feelings of being cared for and protected. Hence, attachment-oriented interventions may be very helpful during this pandemic, as clinicians need to become aware of the emotional and behavioural strategies that insecurely attached individuals use to cope with distress.

## Data Availability

The datasets used and analyzed during the current study available from the corresponding author on reasonable request.

## Abbreviations

RQ: Relationship Questionnaire
RULS: Revised UCLA Loneliness Scale
C-19ASS: The COVID-19 Anxiety Syndrome Scale
GHQ-12: The General Health Questionnaire
